# 肺部肿瘤射频消融治疗的临床应用与进展

**DOI:** 10.3779/j.issn.1009-3419.2010.11.13

**Published:** 2010-11-20

**Affiliations:** 528403 中山，中山市人民医院（中山大学附属中山医院）放射影像中心介入室 Department of Interventional Radiology, Zhongshan City Peoples' Hospital, Zhongshan 528403, China

**Keywords:** 肺部肿瘤, 射频消融, 临床应用, Lung neoplasms, Radiofrequency ablation, Clinical application

## Abstract

肺癌是全世界因肿瘤死亡的首位原因。近年来，射频消融作为一种微创治疗方法在原发性和继发性肺部肿瘤治疗中得到越来越多的应用，并取得了较大进展。射频消融术后的疗效评价并不简单，推荐使用CT、MRI和PET综合评价。本文对其原理、基础研究、临床应用、疗效、进展等方面进行综述。

## Abstract (en)

Lung cancer is the leading cause of cancer-related mortality worldwide. In recent years, radiofrequency ablation (RFA), as a minimally invasive therapy, has been increasing utilized as a non-surgical treatment option for patients with primary and metastatic lung tumors, and great advances have been achieved. The assessment of response after RFA is challenging, and we recommend the use of CT scans, MRI scans and PET scans to assess response rate. The purpose of the article is to review the principles, experimental background, clinical application and response in the treatment of lung neoplasms.

目前，肺癌是人类因肿瘤死亡最常见的原因之一。根据肺癌的生物学特性与治疗方法的不同，肺癌被分成小细胞肺癌（small cell lung cancer, SCLC）和非小细胞肺癌（non-small cell lung cancer, NSCLC）两类，其中NSCLC占75%-80%，治疗采用以手术治疗为主的综合治疗，但包括手术在内的多种治疗方法疗效皆不尽人意。而且80%的肺癌患者在明确诊断时已失去手术机会，这时需要有其它治疗方法来替代手术治疗。同时，肺脏是实体肿瘤第二位易转移的器官，紧跟在淋巴系统之后^[1]^。尸检显示，多达25%-30%的肿瘤患者有肺转移^[2]^。外科手术治疗常用于转移瘤数量有限及局限的患者，且手术需要切除一部分有正常功能的肺组织，因此，也需要新的治疗手段来替代手术。而最近二十年发展起来的射频消融（radiofrequency ablation, RFA）技术有望能扮演这一角色。自从2000年Dupuy等^[3]^首先报道应用经皮射频消融治疗肺部肿瘤后，此技术已经在国内外广泛开展应用，并取得了很大进展。

## 1 射频消融原理

射频消融术通过插入肿瘤组织中的电极针和患者大腿表面粘贴的电极板构成电流回路，开启射频发生器之后，电极尖端的高频交流电射入靶组织，使组织中的离子发生震荡，随之摩擦生热，使电极周围的靶组织内细胞死亡并发生凝固坏死，同时使肿瘤周围的血管组织凝固形成一个反应带，使之不能向肿瘤继续供血，并且可防止肿瘤转移。研究^[4]^表明，当局部温度达50 ℃时持续5 min以上，细胞发生凝固性坏死；60 ℃以上细胞内蛋白质迅速凝固，细胞膜破裂或溶解，细胞内的溶酶体、线粒体、蛋白质、DNA发生不可逆的变性，这样就达到了局部消融肿瘤的目的。治疗性射频消融加热组织的温度范围通常在60 ℃-100 ℃，可导致蛋白变质、酶失活，使细胞瞬间坏死。射频波还可使肿瘤局部血管凝固，血供减少。同时消融后的肿瘤组织存留在体内，由于其成分及结构改变，可刺激机体免疫力并产生抗肿瘤性细胞毒性抗体，并诱导细胞毒性T细胞免疫。

影响射频消融的主要因素^[5]^包括：①血流灌注程度；②电流和热量的传导性；③热敏感程度和是否有其它辅助治疗。

## 2 动物模型及实验研究

Goldberg等^[6]^注射VX2肉瘤细胞悬液至11只兔子右下肺建立肿瘤模型，7个肿瘤行RFA，4个肿瘤作为对照。研究发现RFA后立即行CT复查显示有圆形混浊影包围肿瘤，病理学验证是凝固坏死的肿瘤及外周的肺泡。其中至少95%的经治疗的肿瘤结节在病检时发现有坏死，但3只（43%）兔子发现病灶周边有残余存活肿瘤。作为对照的4只兔子在尸检时发现肿瘤仍继续生长而无坏死（平均存活23 d）。作者认为经皮RFA能成功治疗动物模型中的实体肿瘤结节。Miao等^[7]^将18只肺内种植VX2肿瘤的兔子分为经RFA治疗组（*n*=12）和对照组（*n*=6）进行观察，3个月后对照组兔子全部死亡，治疗组9只（75.0%）获得肿瘤根除，1只肿瘤消融不完全，2只局部肿瘤复发和（或）肺转移。两组比较3个月生存率有统计学差异（*P* < 0.01）。典型MRI表现为急性RFA损伤，包括5个典型等中心区：针道区（A区）、肿瘤凝固区（B区）、肺实质凝固区（C区）、外周出血区（D区）、炎性层区（E区）。Clasens等^[8]^在电镜下发现肿瘤及周边组织中有象征细胞凋亡的凋亡小体存在，表明治疗组织有凝固性坏死。张卫强等^[9]^发现毁损灶的病理改变分为三个阶段：炎症反应期、纤维增生期、结构恢复期。其又取新西兰白兔20只，分为两组。A组将电极置于右肺门，B组置于右下肺外周部位。RFA术后，观察发现A组治疗时间、阻抗与B组比较均有统计学差异。作者分析是由于肺门部血流丰富，带走了部分热量，导致阻抗变低，治疗时间延长。实验中6只兔子发生肺部感染，2只兔子损毁灶形成脓肿，故提醒术者要注意无菌操作，术后应予抗生素预防感染。李虹义等^[10]^研究发现随着支气管腔的变小，射频对支气管的损伤逐渐加重，而对主支气管、二级支气管损伤较轻。

## 3 适应症和禁忌症

### 3.1 适应症

① Ⅰ期或Ⅱa期NSCLC不宜手术者；②Ⅲb期（同一肺叶内出现卫星结节）或Ⅳ期（其它肺叶或另一肺出现结节）NSCLC不宜手术者；③Ⅲa期或Ⅳ期肺癌标准治疗后残余孤立性结节者；④已控制或可控制原发疾病的肺转移瘤且不适宜手术者；⑤靶病变≤5 cm^[11]^。

### 3.2 禁忌症

① 肿瘤紧靠肺门或大肺血管；②恶性胸腔积液；③肺动脉高压；④同一肺中肿瘤数目>3个；⑤靶病变>5 cm^[11]^。

## 4 技术要点

术前通常先对患者进行评估，注意有无心肺功能不全、出血史、感染、使用抗凝血药及支气管扩张剂等。应有近期的影像学检查，如CT或PET/CT，从而评估肿瘤性质、大小、邻近结构等。影像学检查对制定治疗计划十分重要，如设定进针路径、选择消融针的种类及合适的影像引导方式。射频消融常见的治疗途径有：①X线、B超、CT引导下经皮射频消融；②开胸术中射频消融；③经胸腔镜射频消融。

术前可进行全身麻醉或局部麻醉，如采用局麻，要麻醉充分，使消融针所经肺组织肺泡内完全实变，可减少气胸发生率。要注意正确连接电极片，减少皮肤被灼伤的风险。如为CT引导，还要注意根据病灶所在肺内的位置调整患者在检查床上的位置，留出足够的空间以方便术者操作及CT扫描。选择适当的穿刺部位、方向、深度，使电极针最大程度包绕肿瘤组织，肿瘤直径>5 cm病灶注意多点、多面、分次灭活，提高毁损成功率^[12]^。术后应常规使用抗生素，注意气胸、咯血、肺炎等并发症，必要时采取相应治疗措施。要注意的是，如果病灶邻近纵隔，行RFA可能会损伤神经结构如喉返神经或迷走神经，同样在治疗肺尖部肿瘤时可能会损伤臂丛神经。由于有损伤心脏、大血管、中央气道、食道、神经、心包膜的风险，当肿瘤位于纵隔时不宜行RFA治疗。

## 5 疗效及疗效的影像评价

### 5.1 疗效

对比外科手术，消融治疗能部分达到外科手术的治疗目的，且住院时间短，并发症相对较轻，对肺功能无损害，并能对复发和新出现的病灶进行重复治疗。2001年7月-2005年12月在欧洲、美国及澳大利亚的一项前瞻性、多中心临床研究^[13]^中，106例患者共183个肺部肿瘤在CT引导下行RFA治疗，其中NSCLC 33例，结直肠癌肺转移53例，其它部位恶性肺转移瘤20例。患者持续1年的靶肿瘤完全缓解（complete response, CR）率为88%（75/88）。NSCLC及肺部转移瘤疗效无明显差异。NSCLC患者1年和2年总生存（overall survival）率分别为70%和48%；结直肠癌肺转移1年和2年总生存率分别为89%和66%；其它部位恶性肺转移瘤1年和2年总生存率分别为92%和64%。NSCLC患者1年和2年肿瘤专项生存（cancer-specific survival）率分别为92%和73%；结直肠癌肺转移患者1年和2年肿瘤专项生存率分别为91%和68%；其它部位恶性肺转移瘤1年和2年肿瘤专项生存率分别为93%和67%。Ⅰ期NSCLC 2年总生存率为75%，2年肿瘤专项生存率为92%。Zemlyak等^[14]^回顾了2003年-2008年共64例不能行标准切除术的Ⅰ期NSCLC患者治疗情况，其中25例行局限性切除（sublobar resections, SLR），12例行RFA，27例行经皮冷冻消融（pe rcut aneous cryoablation therapy, PCT）。SLR、RFA和PCT组3年生存率分别为87.1%、87.5%、77%（P>0.05）。SLR、RFA和PCT组的3年肿瘤专项生存率分别为90.6%、87.5%、50%，3年无癌生存（cancer-free sur vival）率分别为60.8%、50%、50%。

射频消融还可以用来缓解晚期肺癌患者的各种不适症状。Lee等^[15]^报道了20例晚期NSCLC患者行RFA后，80%咯血的患者、30%胸痛的患者、36%呼吸困难的患者和25%咳嗽的患者症状都得到了一定改善。Vansonnenberg等^[16]^随访发现RFA治疗可缓解疼痛。

### 5.2 疗效的影像评价

在临床中，RFA术后疗效的评价比较困难。在不同文献中，怎样去评价和定义疗效有较大的争议。

#### 5.2.1 CT评价

治疗后即刻复查CT最典型的影像表现是在消融区出现一个圆形磨玻璃样密度影，范围比术前病灶要大，CT值亦减低，可见蜂窝状低密度影（微小气泡影）。在术后1个月-3个月期间，消融区域普遍出现空洞，这些空洞随着时间延长通常会收缩。在此期间，透明泡沫影（1 mm-3 mm）能被观察到，亦随时间而消散。一些病灶在术后1个月-3个月期间会变大，此后会变得稳定。6个月后病灶及空洞的大小相对无变化，6个月后病灶增大则代表局部复发^[17]^。病灶大小变化是评价疗效的一个关键指标，但有学者指出，RFA能导致病灶周围区域组织出现炎症，术后初期病灶范围会增大，之后范围会缩小，因此术后早期利用大小作为评价疗效的标准可能会不准确。一些学者指出，术后30 d时消融区周围炎症层和出血层通常会恢复到最初的病灶大小^[18]^。影响评价的另一个中心问题是如何判别消融后病灶区是否有残余存活肿瘤。Jin^[19]^提出CT图像在消融结束时行增强CT时被消融区不应该有任何增强。Lee等^[20]^认为增强前和增强后10 HU CT值的增加则代表存活未被消融的肿瘤。而Sharma等^[21]^通过CT随访观察到RFA术后第一个月可伴随肺门和纵隔局部淋巴结肿大，但在RFA术后前6个月可逐渐减小，这可能与消融引起的肺部炎症有关，此时不应误解为淋巴结转移。此外，少数病例中增大的淋巴结可能有FDG摄取量增加或在增强CT中有增强。

虽然CT在RFA术后随访中起着举足轻重的作用，但是单独应用CT评价疗效是不够的。Belfiore^[22]^发现CT评价容易出现假阴性，一些消融区的活检标本中病检证实有瘤细胞存在，甚至是在病灶区减小的病例中被发现。

#### 5.2.2 MRI评价

Oyama等^[23]^通过建立动物模型，利用MRI随访RFA治疗效果，结果见表 1。

**1:** 肺部病变射频消融后MRI和组织病理学改变摘要 Summary of the MRI and histopathologic findings of radiofrequency-ablated lesions in the lung

Phase	Tl-weighted imaging	T2-weighted imaging	Contrast-enhanced T1-weighted imaging	Histopathologic findings
Acute				
Inner zone	Isointensity	Hypointensity	Non-enhancement	Eosinophilic-staining tissue with pyknotic nuclei
Outer zone	Isointensity	Hyperintensity	Ring-like enhancement	Neutrophilic infiltration, congestion, alveolar fluid collections
Subacute				
Inner zone	Hyperintensity	Hypointensity	Non-enhancement	Coagulative necrosis
Outer zone	Isointensity	Hyperintensity	Ring-like enhancement	Granulation with inflammatory infiltration
Chronic				
Inner zone	Hyperintensity	Heterogeneous intensity	Non-enhancement	Coagulative necrosis
Outer zone	Isointensity or hyperintensity	Hyperintensity	Ring-like enhancement	Thickened fibrous tissue with inflammatory cells
Reproduced with permission from Oyama Y, Nakamura K, Matsuoka T, *et al*. Radiofrequency ablated lesion in the normal porcine lung: long-term follow-up with MRI and pathology. Cardiovasc Intervent Radiol, 2005, 28(3): 346-353.				

另一项前瞻性实验研究^[24]^发现局部无进展病灶的表面弥散系数（apparent diffusion coefficient, ADC）值明显高于术后局部复发病灶ADC值，消融后病灶ADC值明显高于术前，因而可用ADC评价肺肿瘤RFA疗效。

#### 5.2.3 PET

FDG-PET显像可在早期通过测定肿瘤组织代谢变化来判断坏死与存活瘤组织，以此评定疗效。治疗后肿瘤形态的变化往往迟于代谢的变化，而存活瘤组织的代谢通常明显高于坏死瘤组织，所以PET显像可成为鉴别肿瘤良恶性或评价疗效的重要指标^[25]^。

综上所述，评价疗效靠一种检查或一种指标是远远不够的。通常，用于客观评价肿瘤疗效的基于CT或MRI测量病变直径变化的实体肿瘤疗效评价标准（Response Evaluation Criteria in Solid Tumors, RECIST）被广泛使用。按照RECIST标准，疗效分为：完全缓解（complete response, CR）、部分缓解（partial response, PR）、疾病稳定（stable disease, SD）、疾病进展（progressive disease, PD）。Fernando等^[26]^认为一项修正的实体瘤疗效评价标准可以用来评价消融的疗效，定义见表 2。

**2:** 用于评价治疗效果修正的实体瘤疗效评价标准 Modified RECIST criteria used to evaluate treatment response

Response	CT mass size (RECIST)	CT mass quality	PET
Complete (any 2)	Lesion disappearance or scar < 25% original size	Cyst or cavity formation	SUV < 2. 5
Partial (any 1)	Decrease of > 30% in LD of target lesion	Low density of entire lesion Central necrosis or central cavitation with liquid density	Decreased SUV or area of FDG uptake
Stable lesion (any 1)	Decrease of < 30% in LD of target lesion	Mass solid appearance, no Central necrosis or cavity	Unchanged SUV or area of FDG uptake
Progression (any 2)	Increase of > 20% in LD of target lesion	Solid mass, invasion adjacent structures	Higher SUV
Target lesions represent tumors treated with RFA. SUV: standard uptake value of fluorodeoxyglucose F^18^ in PET scan; LD: largest diameter of target lesions; FDG: fluorodeoxyglucose F^18^. Reproduced with permission from Fernando HC, DeHoyos A, Landreneau RJ, *et al*. Radiofrequency ablation for the treatment of non-small cell lung cancer in marginal surgical candidates. J Thorac Cardiovasc Surg, 2005, 129(3): 639-644.			

WHO也推荐了判断RFA术后疾病复发或进展的标准^[27]^：①消融灶在术后3个月-6个月有生长；②消融灶有对比增强：180 s后有>50%基准的增强；>15 mm的结节状增强；任何>15 HU的中心增强；③局部或远处淋巴结肿大和肺内或胸外新发病灶亦代表有进展。

## 6 影响疗效的因素

国内外文献表明疗效可能与肿瘤大小、病理分级、肿瘤分期、邻近结构、肺外转移、CEA水平、年龄、联合其它治疗等因素相关。Chua等^[28]^对100例不宜手术的结直肠癌肺转移的患者行经皮RFA治疗，术后联合辅助全身化疗并观察疗效，单变量分析显示病理分级、RFA术后时间、疗效、重复RFA治疗、肺外转移、纵隔淋巴结肿大和辅助全身化疗与总生存率相关，多变量分析显示疗效、重复RFA治疗、肺外转移和辅助全身化疗是生存率的独立预测因子。另有研究表明>3 cm和 < 3 cm肿瘤对治疗的反应有统计学差异。Akeboshi^[29]^通过31例患者54个肿瘤RFA治疗随访发现，肿瘤 < 3 cm者病灶获完全损毁为69%，肿瘤>3 cm者获完全损毁为39%（*P* < 0.05）。Pennathur等^[30]^发现病变大小是一项重要的与总生存率及无瘤生存率相关的预后性变量（*P* < 0.05）。Gillams等^[31]^发现肿瘤复发普遍存在于大肿瘤中。而Yokouchi^[32]^指出肿瘤复发是因为肿瘤邻近气管、SVC或肺内血管及支气管，原因可能为邻近血管可被血流带走部分热量从而影响消融效果，称为“热沉效应”。Yan等^[33]^研究发现，对于局部无进展生存（local progression-free survival）率，单变量分析显示病灶大小、位置、术后1个月、3个月CEA水平是重要的预后指标，多变量分析显示病灶>3 cm及术后1个月CEA水平>5 ng/mL与局部无进展生存率减小独立相关。

## 7 并发症

射频消融治疗肺部肿瘤的并发症有3类^[34]^：①主要并发症指需要治疗或有不良后果者，如需要胸腔闭式引流的气胸或胸腔积液。②次要并发症是指无需治疗或无不良后果者。③副反应指伴随治疗出现的结果，一般经常发生，但很少造成实际的损害，主要是疼痛。常见的并发症有气胸、胸腔积液、咯血、咳嗽、发热、肺炎、肺脓肿、出血等，其它并发症还有疼痛、皮肤灼伤、皮下气肿、胸膜炎、对慢性阻塞性肺病（chronic obstructive pulmonary disease, COPD）恶化、急性呼吸窘迫综合征（acute respiratory distresssyndrome, ARDS）、血胸、支气管胸膜瘘、声带麻痹、房颤、肺栓子、针道种植等。

Sano等^[35]^通过随访137例患者（366个肿瘤共211次RFA术）发现，2例患者因顽固性气胸和大咯血死亡。总体主要并发症发生率为17.4%（需置管引流的气胸和胸腔积液分别为25例和4例，胸膜炎6例，肺脓肿1例，肺内出血致血胸1例）。次要并发症包括无需引流的气胸和胸腔积液，分别为108例、14例，血痰9例，恶心和（或）呕吐3例，皮下气肿3例，咳嗽2例，皮肤灼伤2例，肺不张1例，肠梗阻1例。Gillams^[36]^指出肿瘤数目、电极针位置、电极针通过充气肺组织路径的长短是影响气胸发生的因素。

## 8 联合治疗

### 8.1 与化疗联合

RFA有较明显的局部治疗效果，使肿瘤细胞凝固坏死，减轻肿瘤负荷，从而增强化疗疗效。同时化疗可对纵隔淋巴结转移的肿瘤细胞产生治疗作用。此外，RFA可改善患者免疫功能，对提高治疗疗效、改善预后可能有一定的临床意义。王少彬等^[37]^将34例晚期NSCLC（Ⅲ期、Ⅳ期）采用RFA联合GP方案治疗，并与单纯GP方案治疗30例NSCLC作比较。结果显示在疗效评价、生存时间、生活质量评分上RFA联合GP方案治疗组明显优于对照组，疗效有统计学差异。

### 8.2 与放疗联合

肿瘤周边的细胞富氧，中央部分细胞则乏氧，富氧有利射线在组织中产生自由基，有助于对细胞的损伤^[38]^，因此肿瘤周边细胞对放疗敏感。射频消融治疗的特点是中心区细胞完全凝固坏死，病灶周边易残存肿瘤细胞。两者联合应用，可弥补各自的不足以增强疗效。Grieco^[39]^认为对于不能手术的Ⅰ/Ⅱ期NSCLC，放疗后联合热消融能使并发症发生率相对较低并容易控制，对比任何一种单独治疗模式，联合治疗能改善生存率。

### 8.3 与介入灌注联合

孙一等^[40]^对观察组32例晚期NSCLC患者行RFA治疗，1周后采用含顺铂的方案行支气管动脉灌注化疗，治疗1次-3次；选择患者40例为对照组（同期接受相同化疗方案进行全身化疗的晚期NSCLC），全身化疗2次-4次。结果示观察组和对照组的近期疗效分别为（CR+PR）为78.1%和37.5%，观察组和对照组有统计学差异。随访6个月、12个月、24个月生存率，观察组分别为93.8%、75%和9.4%，对照组分别为67.5%、45%和2.5%，观察组优于对照组。两组毒副作用无统计学差异。

## 9 展望

射频消融是一种局部、微创、安全、可以重复进行的治疗方法，与手术相比，能减少对正常肺组织的损伤，避免开胸手术对患者的打击，减少手术相关发病率和死亡率，并且花费低、住院时间短。射频消融对高风险、不能手术的NSCLC及肺转移瘤在很大程度上能起到代替手术的作用。目前，在评估射频消融疗效方面还存在争议，在这里我们推荐用修正的实体瘤评价标准。术后复发率高、对大肿瘤及特殊位置肿瘤疗效欠佳是当前射频消融存在的问题，但是随着技术的进步和经验的累积，射频消融必将在肺部肿瘤的治疗中有着更加广阔的前景。

## References

[1] Willis RA (1973). The spread of tumours in the human body.

[2] Ollila DW, Morton DL (1998). Surgical resection as the treatment of choice for melanoma metastatic to the lung. Chest Surg Clin N Am.

[3] Dupuy DE, Zagoria RJ, Akerley W (2000). Percutaneous radiofrequency ablation of malignancies in the lung. AJR.

[4] Goldberg SN, Dupuy DE (2001). Image-guided radiofrequency tumor ablation: challenges and opportunities--part Ⅰ. J Vasc Interv Radiol.

[5] Chen MH, Golgberg SN (2009). Radiofrequency ablation of liver tumors-basic and clinical studies.

[6] Goldberg SN, Gazelle GS, Compton CC (1996). Radio-frequency tissue ablation of VX2 tumor nodules in the rabbit lung. Acad Radiol.

[7] Miao Y, Ni Y, Bosmans H (2001). Radiofrequency ablation for eradication of pulmonary tumor in rabbits. J Surg Res.

[8] Clasen S, Krober SM, Kosan B (2008). Pathomorphologic evaluation of pulmonary radiofrequency ablation: proof of cell death is characterized by DNA fragmentation and apoptotic bodies. Cancer.

[9] Zhang WQ, Cheng QS, Ma LJ (2002). The biological effects of multi-electrode radiofrequency ablation on normal rabbit lungs. Chin J Lung Cancer.

[10] Li HY, Zhu WL, Wan YH (2008). Influence of radiofrequency ablation on different grades of bronchus. J Fourth Mil Med Univ.

[11] Abbas G, Pennathur A, Landreneau RJ (2009). Radiofrequency and microwave ablation of lung tumors. J Surg Oncol.

[12] Liu BD, Zhi XY, Liu L (2009). Evaluation of three-dimensional reconstruction ct in percutaneous radiofrequency ablation (RFA) of the unresectable lung tumor with a clustered electrode. Chin J Lung Cancer.

[13] Lencioni R, Crocetti L, Cioni R (2008). Response to radiofrequency ablation of pulmonary tumours: a prospective, intention-to-treat, multicentre clinical trial (the RAPTURE study). Lancet Oncol.

[14] Zemlyak A, Moore WH, Bilfinger TV (2010). Comparison of survival after sublobar resections and ablative therapies for stage Ⅰ non-small cell lung cancer. J Am Coll Surg.

[15] Lee JM, Jin GY, Goldberg SN (2004). Percutaneous radiofrequency ablation for inoperable non-small cell lung cancer and metastases: preliminary report. Radiology.

[16] VanSonnenberg E, Shankar S, Morrison PR (2005). Radiofrequency ablation of thoracic lesions: part 2, initial clinical experience--technical and multidisciplinary considerations in 30 patients. AJR.

[17] Bojarski JD, Dupuy DE, Mayo-Smith WW (2005). CT imaging findings of pulmonary neoplasms after treatment with radiofrequency ablation: results in 32 tumors. AJR.

[18] Nguyen CL, Scott WJ, Goldberg M (2006). Radiofrequency ablation of lung malignancies. Ann Thorac Surg.

[19] Jin GY, Lee JM, Lee YC (2004). Primary and secondarylung malignancies treated with percutaneous radiofrequency ablation: evaluation with followup helical CT. AJR.

[20] Lee JM, Jin GY, Goldberg SN (2004). Percutaneous radiofrequency ablation for inoperable nonsmall cell lung cancer and metastases: Preliminary report. Radiology.

[21] Oyama Y, Nakamura K, Matsuoka T (2010). Reversible locoregional lymph node enlargement after radiofrequency ablation of lung tumors. AJR.

[22] Belfiore1 G, Moggio1 G, Tedeschi1 E (2004). CT-guided radiofrequency ablation: a potential complementary therapy for patients with unresectable primary lung cancer--a preliminary report of 33 patients. AJR.

[23] Oyama Y, Nakamura K, Matsuoka T (2005). Radiofrequency ablated lesion in the normal porcine lung: long-term follow-up with MRI and pathology. Cardiovasc Interv Radiol.

[24] Okuma T, Matsuoka T, Yamamoto A (2009). Assessment of early treatment response after CT-guided radiofrequency ablation of unresectable lung tumours by diffusion-weighted MRI: a pilot study. Br J Radiol.

[25] Lowe VJ, Fletcher JW, Gobar L (1998). Prospective investigation of positron emission tomography in lung nodules. J Clin Oncol.

[26] Fernando HC, DeHoyos A, Landreneau RJ (2005). Radiofrequency ablation for the treatment of non-small cell lung cancer in marginal surgical candidates. J Thorac Cardiovasc Surg.

[27] Casal RF, Tam AL, Eapen GA (2010). Radiofrequency ablation of lung tumors. Clin Chest Med.

[28] Chua TC, Thornbury K, Saxena A (2010). Radiofrequency ablation as an adjunct to systemic chemotherapy for colorectal pulmonary metastases. Cancer.

[29] Akeboshi M, Yamakado K, Nakatsuka A (2004). Percutaneous radiofrequency ablation of lung neoplasm: initial therapeutic response. J Vasc Interv Radiol.

[30] Pennathur A, Abbas G, Qureshi I (2009). Radiofrequency ablation for the treatment of pulmonary metastases. Ann Thorac Surg.

[31] Gillams AR, Lees WR (2008). Radiofrequency ablation of lung metastases: factors influencing success. Eur Radiol.

[32] Yokouchi H, Yasumoto T, Murata K (2008). Radiofrequency ablation of malignant lung tumors--preliminary report of 12 cases. Gan To Kagaku Ryoho.

[33] Yan TD, King J, Sjarif A (2007). Treatment failure after percutaneous radiofrequency ablation for nonsurgical candidates with pulmonary metastases from colorectal carcinoma. Ann Surg Oncol.

[34] Zhi XY, Liu BD (2009). Advances and current situation in radiofrequency of lung cancer. Chin J Clinicians (Electronic Edition).

[35] Sano Y, Kanazawa S, Gobara H (2007). Feasibility of percutaneous radiofrequency ablation for intrathoracic malignancies: a large single-center experience. Cancer.

[36] Gillams AR, Lees WR (2007). Analysis of the factors associated with radiofrequency ablation-induced pneumothorax. Clin Radiol.

[37] Wang SB, Chen JH, Cao WH (2005). The observation of the clinical effect for combination therapy of RFA with GP on advanced stage lung cancer. Chin J Clin Oncol.

[b38] 38Wan DS ed. Clinical Oncology. 2nd ed. Beijing: Science Press, 2006. 277-278.万德森主编. 临床肿瘤学. 第2版. 北京: 科学出版社, 2006. 277-288.

[39] Grieco CA, Simon CJ, Mayo-Smith WW (2006). Percutaneous image-guided thermal ablation and radiation therapy: outcomes of combined treatment for 41 patients with inoperable stage Ⅰ/Ⅱ non-small-cell lung cancer. J Vasc Interv Radiol.

[40] Su nY, Dong Y, Xiao P (2009). Clinical value of radiofrequency ablation combined with bronchial artery infusion chemotherapy for the late nonsmall lung cancer. J Mini Invas Med.

